# Risk Factors for Severe Outcomes following 2009 Influenza A (H1N1) Infection: A Global Pooled Analysis

**DOI:** 10.1371/journal.pmed.1001053

**Published:** 2011-07-05

**Authors:** Maria D. Van Kerkhove, Katelijn A. H. Vandemaele, Vivek Shinde, Giovanna Jaramillo-Gutierrez, Artemis Koukounari, Christl A. Donnelly, Luis O. Carlino, Rhonda Owen, Beverly Paterson, Louise Pelletier, Julie Vachon, Claudia Gonzalez, Yu Hongjie, Feng Zijian, Shuk Kwan Chuang, Albert Au, Silke Buda, Gerard Krause, Walter Haas, Isabelle Bonmarin, Kiyosu Taniguichi, Kensuke Nakajima, Tokuaki Shobayashi, Yoshihiro Takayama, Tomi Sunagawa, Jean Michel Heraud, Arnaud Orelle, Ethel Palacios, Marianne A. B. van der Sande, C. C. H. Lieke Wielders, Darren Hunt, Jeffrey Cutter, Vernon J. Lee, Juno Thomas, Patricia Santa-Olalla, Maria J. Sierra-Moros, Wanna Hanshaoworakul, Kumnuan Ungchusak, Richard Pebody, Seema Jain, Anthony W. Mounts, on behalf of the WHO Working Group for Risk Factors for Severe H1N1pdm Infection

**Affiliations:** 1 Global Influenza Programme, World Health Organization; 2 Medical Research Council Centre for Outbreak Analysis and Modelling, Department of Infectious Disease Epidemiology, Imperial College London, London, United Kingdom; 3 Ministerio de Salud de la Nación, Buenos Aires, Argentina; 4 Influenza Surveillance Section, Surveillance Branch, Office of Health Protection, Department of Health and Ageing, Woden, Australia; 5 Influenza Surveillance Section, Public Health Agency of Canada, Ontario, Canada; 6 Departamento de Epidemiología, División de Planificación Sanitaria, Ministerio de Salud de Chile, Santiago, Chile; 7 Office for Disease Control and Emergency Response, Chinese Center for Disease Control and Prevention Beijing, China; 8 Surveillance and Epidemiology Branch, Centre for Health Protection, Centre for Health Protection of Department of Health, Hong Kong; 9 Department for Infectious Disease Epidemiology, Robert Koch Institute, Berlin, Germany; 10 Département des Maladies Infectieuses, Institut de Veille, Sanitaire, Saint-Maurice Cedex, France; 11 Infectious Disease Surveillance Center, National Institute of Infectious Diseases, Tokyo, Japan; 12 Ministry of Health, Labour and Welfare, Tokyo, Japan; 13 Virology Unit, Institut Pasteur from Madagascar, Antananarivo, Madagascar; 14 Directorate General of Epidemiology, Mexico City, Mexico; 15 Epidemiology and Surveillance Unit, Centre for Infectious Disease Control, National Institute for Public Health and the Environment, Bilthoven, the Netherlands; 16 New Zealand Ministry of Health, Wellington, New Zealand; 17 Communicable Diseases Division at the Ministry of Health, Singapore; 18 Biodefence Centre, Ministry of Defence, Singapore; 19 Department of Epidemiology and Public Health, Yong Loo Lin School of Medicine, National University of Singapore, Singapore; 20 Epidemiology and Surveillance Unit, Respiratory Virus Unit, National Institute for Communicable Diseases, National Health Laboratory Service, Johannesburg, South Africa; 21 Coordinating Centre for Health Alerts and Emergencies, Dirección General de Salud Pública y Sanidad Exterior Ministerio de Sanidad y Política Social, Madrid, Spain; 22 Department of Disease Control, Ministry of Public Health, Nonthaburi, Thailand; 23 Health Protection Agency, London, United Kingdom; 24 Epidemiology and Prevention Branch, Influenza Division, Centers for Disease Control and Prevention, Atlanta, Georgia, United States of America; The University of Hong Kong, Hong Kong

## Abstract

**Background:**

Since the start of the 2009 influenza A pandemic (H1N1pdm), the World Health Organization and its member states have gathered information to characterize the clinical severity of H1N1pdm infection and to assist policy makers to determine risk groups for targeted control measures.

**Methods and Findings:**

Data were collected on approximately 70,000 laboratory-confirmed hospitalized H1N1pdm patients, 9,700 patients admitted to intensive care units (ICUs), and 2,500 deaths reported between 1 April 2009 and 1 January 2010 from 19 countries or administrative regions—Argentina, Australia, Canada, Chile, China, France, Germany, Hong Kong SAR, Japan, Madagascar, Mexico, the Netherlands, New Zealand, Singapore, South Africa, Spain, Thailand, the United States, and the United Kingdom—to characterize and compare the distribution of risk factors among H1N1pdm patients at three levels of severity: hospitalizations, ICU admissions, and deaths. The median age of patients increased with severity of disease. The highest per capita risk of hospitalization was among patients <5 y and 5–14 y (relative risk [RR] = 3.3 and 3.2, respectively, compared to the general population), whereas the highest risk of death per capita was in the age groups 50–64 y and ≥65 y (RR = 1.5 and 1.6, respectively, compared to the general population). Similarly, the ratio of H1N1pdm deaths to hospitalizations increased with age and was the highest in the ≥65-y-old age group, indicating that while infection rates have been observed to be very low in the oldest age group, risk of death in those over the age of 64 y who became infected was higher than in younger groups. The proportion of H1N1pdm patients with one or more reported chronic conditions increased with severity (median = 31.1%, 52.3%, and 61.8% of hospitalized, ICU-admitted, and fatal H1N1pdm cases, respectively). With the exception of the risk factors asthma, pregnancy, and obesity, the proportion of patients with each risk factor increased with severity level. For all levels of severity, pregnant women in their third trimester consistently accounted for the majority of the total of pregnant women. Our findings suggest that morbid obesity might be a risk factor for ICU admission and fatal outcome (RR = 36.3).

**Conclusions:**

Our results demonstrate that risk factors for severe H1N1pdm infection are similar to those for seasonal influenza, with some notable differences, such as younger age groups and obesity, and reinforce the need to identify and protect groups at highest risk of severe outcomes.

*Please see later in the article for the Editors' Summary*

## Abstract 2

This study analyzes data from 19 countries (from April 2009 to Jan 2010), comprising some 70,000 hospitalized patients with severe H1N1 infection, to reveal risk factors for severe pandemic influenza, which include chronic illness, cardiac disease, chronic respiratory disease, and diabetes.

## Editors' Summary

**Background:**

In April 2009, a new strain of influenza A H1N1 was first identified in Mexico and the United States and subsequently spread around the world. In June 2009, the World Health Organization (WHO) declared a pandemic alert phase 6, which continued until August 2010. Throughout the pandemic, WHO and member states gathered information to characterize the patterns of risk associated with the new influenza A H1N1 virus infection and to assess the clinical picture. Although risk factors for severe disease following seasonal influenza infection have been well documented in many countries (for example, pregnancy; chronic medical conditions such as pulmonary, cardiovascular, renal, hepatic, neuromuscular, hematologic, and metabolic disorders; some cognitive conditions; and immunodeficiency), risk factors for severe disease following infection early in the 2009 H1N1 pandemic were largely unknown.

**Why Was This Study Done?:**

Many countries have recently reported data on the association between severe H1N1 influenza and a variety of underlying risk factors, but because these data are presented in different formats, making direct comparisons across countries is difficult, with no clear consensus for some conditions. Therefore, to assess the frequency and distribution of known and new potential risk factors for severe H1N1 infection, this study was conducted to collect data (from 1 April 2009 to 1 January 2010) from surveillance programs of the Ministries of Health or National Public Health Institutes in 19 countries―Argentina, Australia, Canada, Chile, China, France, Germany, Hong Kong (special administrative region), Japan, Madagascar, Mexico, the Netherlands, New Zealand, Singapore, South Africa, Spain, Thailand, the United States, and the United Kingdom.

**What Did the Researchers Do and Find?:**

As part of routine surveillance, countries were asked to provide risk factor data on laboratory-confirmed H1N1 in patients who were admitted to hospital, admitted to the intensive care unit (ICU), or had died because of their infection, using a standardized format. The researchers grouped potential risk conditions into four categories: age, chronic medical illnesses, pregnancy (by trimester), and other conditions that were not previously considered as risk conditions for severe influenza outcomes, such as obesity. For each risk factor (except pregnancy), the researchers calculated the percentage of each group of patients using the total number of cases reported in each severity category (hospitalization, admission to ICU, and death). To evaluate the risk associated with pregnancy, the researchers used the ratio of pregnant women to all women of childbearing age (age 15–49 years) at each level of severity to describe the differences between levels.

The researchers were able to collect data on approximately 70,000 patients requiring hospitalization, 9,700 patients admitted to the ICU, and 2,500 patients who died from H1N1 infection. The proportion of patients with H1N1 with one or more reported chronic conditions increased with severity—the median was 31.1% of hospitalized patients, 52.3% of patients admitted to the ICU, and 61.8% of patients who died. For all levels of severity, pregnant women in their third trimester consistently accounted for the majority of the total of pregnant women. The proportion of patients with obesity increased with increasing disease severity—median of 6% of hospitalized patients, 11.3% of patients admitted to the ICU, and 12.0% of all deaths from H1N1.

**What Do These Findings Mean?:**

These findings show that risk factors for severe H1N1 infection are similar to those for seasonal influenza, with some notable differences: a substantial proportion of people with severe and fatal cases of H1N1 had pre-existing chronic illness, which indicates that the presence of chronic illness increases the likelihood of death. Cardiac disease, chronic respiratory disease, and diabetes are important risk factors for severe disease that will be especially relevant for countries with high rates of these illnesses. Approximately 2/3 of hospitalized people and 40% of people who died from H1N1 infection did not have any identified pre-existing chronic illness, but this study was not able to comprehensively assess how many of these cases had other risk factors, such as pregnancy, obesity, smoking, and alcohol misuse. Because of large differences between countries, the role of risk factors such as obesity and pregnancy need further study—although there is sufficient evidence to support vaccination and early intervention for pregnant women. Overall, the findings of this study reinforce the need to identify and target high-risk groups for interventions such as immunization, early medical advice, and use of antiviral medications.

**Additional Information:**

Please access these Web sites via the online version of this summary at http://dx.doi.org/10.1371/journal.pmed.1001053.

- WHO provides a Global Alert and Response (GAR) with updates on a number of influenza-related topics
- The US Centers for Disease Control and Prevention provides information on risk factors and H1N1

## Introduction

In late April 2009, a novel strain of influenza A H1N1 was identified in Mexico and the United States. This virus quickly spread globally, and on June 11, 2009, the World Health Organization (WHO) declared a pandemic alert phase 6, indicating that the first influenza pandemic of the 21^st^ century had begun [1]–[3]. Many Northern hemisphere temperate countries experienced their first wave of infection during the spring and summer months of 2009, followed by an early 2009 fall influenza season. Southern hemisphere temperate countries experienced the first wave of infection during their winter of 2009, and at the time of writing are finishing their winter 2010 season. By the end of 2009, the peak of the local influenza epidemic had passed in most countries around the world [4].

Since the start of the pandemic, WHO and member states have been gathering information to characterize the clinical picture and patterns of risk associated with the 2009 pandemic influenza A H1N1 (H1N1pdm) virus infection to assist public health policy makers in targeting of vaccination strategies, antiviral use, and other control measures. Risk factors for severe disease following seasonal influenza infection have been well documented in many countries, and include chronic medical conditions such as pulmonary, cardiovascular, renal, hepatic, neuromuscular, hematologic, and metabolic disorders, some cognitive conditions, and immunodeficiency [5]–[7]. The risk associated with seasonal influenza during pregnancy is less well documented but in previous pandemics, pregnant women were identified as being at increased risk of adverse outcomes, and many countries include healthy pregnant women among the seasonal influenza high risk groups as well [8]–[11]. However, early in the 2009 H1N1 pandemic, risk factors for severe disease following infection were largely unknown. Following a series of teleconferences organized by WHO with clinicians treating H1N1pdm patients around the world, it appeared that the most common risk factors for severe H1N1pdm disease were similar to those for seasonal influenza infection; however, several new factors (e.g., obesity and tuberculosis [TB]) were also observed with high frequency in some countries. It was also noted that members of indigenous/aboriginal communities in some countries appeared to be overrepresented among severe cases [12].

While many countries have recently reported data on the association between severe H1N1pdm influenza and the presence of a variety of underlying risk factors (e.g., [13]–[26]), these data are presented in different formats, making direct comparisons across countries difficult, and no clear consensus has emerged for some conditions. This paper presents data from approximately 70,000 lab-confirmed hospitalized and 2,500 fatal cases of H1N1pdm infection in 19 countries or administrative regions—Argentina, Australia, Canada, Chile, China, France, Germany, Hong Kong SAR, Japan, Madagascar, Mexico, the Netherlands, New Zealand, Singapore, South Africa, Spain, Thailand, the United States, and the United Kingdom—in order to characterize and compare the distribution of underlying risk factors among H1N1pdm confirmed patients who were hospitalized, admitted to an intensive care unit (ICU), or died, and to assess the frequency and distribution of known and new potential risk factors for severe H1N1pdm infection.

## Methods

This study compares data primarily obtained from surveillance programs of the Ministries of Health or National Public Health Institutes of 19 countries or administrative regions covering the period 1 April 2009 to 1 January 2010. Countries were asked to provide risk factor data on laboratory-confirmed cases using a standardized format for this analysis. The data were collected in the course of routine surveillance, methods of which varied from country to country [27]–[49], and were reported anonymously and as aggregate data; hence, no ethics approval was required. It should be noted that considerable effort was put into negotiating permission for these data to be presented in these formats. As many countries would not be willing to have their country-specific data published in direct comparisons with others, we are taking the approach of publishing data from a wide range of countries and showing the variability observed, so that results from specific studies can be compared with the international results reported here.

Potential risk factors were grouped into four categories: age, chronic medical illnesses, pregnancy (by trimester), and “other,” which included conditions that were not previously considered as risk factors for severe influenza outcomes, such as obesity, membership in a vulnerable social or ethnic group, and TB. Details of the standardized format and definitions of each of the conditions are provided in Text S1.

Risk factor information was collected separately for three levels of severity of illness in laboratory-confirmed patients: hospitalizations, admissions to ICU, and fatalities by country. Details of the available data from countries by risk factor and severity level are provided in . For each risk factor, except for pregnancy, the percentage of patients who were hospitalized, were admitted to ICU, and died was calculated using the total number of cases reported in each severity category. To evaluate the risk associated with pregnancy, the ratio of pregnant women to all women of childbearing age (age 15–49 y) in each level of severity was used to describe the differences between levels. The overall median and interquartile ranges (IQRs) were calculated for each risk factor using all available data. In addition, where available, countries provided baseline comparison data for prevalence of the risk factor in the general population (details and sources provided in Text S1). Data on age were provided by age groups (<5, 5–14, 15–24, 25–49, 50–64, and ≥65 y).

### Risk of Severe Disease

Where data were available, we calculated the risk for severe H1N1pdm outcomes (hospitalization, admission to ICU, and death) compared to the prevalence of risk factors in the general population (relative risk [RR] of hospitalization [RR_hosp_], RR of ICU admission [RR_ICU_], and RR of death [RR_death_]) by country. See Text S1 for more information and formulae.

For pregnancy, we first calculated the proportion of women of childbearing age who were pregnant in each severity category by dividing the number of pregnant women in that category by the number of women of childbearing age in that category. As individual case data were not available, we calculated the number of fertile women in each level of severity using the numbers of patients in each level of severity in the age range between 15 and 49 y multiplied by the percentage for that severity level that was female. Unless provided by the country, the point prevalence of pregnant women in the general population (the denominator of the RR calculation) was calculated using crude birth rate and 2010 United Nations population estimates [50] to derive the annual number of pregnancies, multiplied by 40/52 and without adjusting for seasonality of pregnancies, abortions, miscarriages, early deliveries, or multiple births. We also calculated the country-specific odds ratios (ORs) and 95% confidence intervals (CIs) for death given hospitalization separately for each risk factor (i.e., the odds of death given hospitalization and a specific risk factor), thereby comparing the odds of death in one group (e.g., among hospitalized patients with asthma) with the odds of death in all other hospitalized patients combined (e.g., among hospitalized patients without asthma) (individual country ORs not shown). We then used the *I* ^2^ statistic to quantify the percentage of variation across countries that is due to true underlying heterogeneity in the ORs rather than chance variability [51]. The *I* ^2^ statistics for all examined risk factors indicated that there was substantial true underlying variation between ORs from different countries. We undertook meta-analyses with and without random effects in parallel to describe the distribution of the OR estimates across the countries for which data were available for analysis. As expected, given the heterogeneity observed between countries, the random effects meta-analysis yielded wider CIs. We conservatively report the pooled estimates from the random effects meta-analysis to describe the distribution of the OR estimates across the countries for which data were available for analysis. Underlying the random effects approach is the assumption that, although the individual countries give rise to different OR estimates, these estimates arise from a distribution with a central value, the estimate of which is referred to as the “pooled OR,” and normally distributed variability around this value. However, because of the limited number of countries in each analysis, the number of random effects is too small for diagnostics such as quantile–quantile plots to demonstrate whether the assumption of a normal distribution is valid.

Finally, with a meta-analysis of data from such diverse countries as those included in this study, reasons for heterogeneity were sought through exploratory meta-regression analyses. However, because of the limited number of countries included in the meta-regression models, and with country being the unit of analysis, the meta-regression results were not considered to be robust and so are not presented or discussed further [52].

All meta-analyses and meta-regression techniques were performed using Stata version 10 (StataCorp).

## Results

Data were collected on approximately 70,000 patients requiring hospitalization, 9,700 patients admitted to ICU, and 2,500 fatalities from 19 countries and administrative regions across the Americas, Asia, Europe, and Africa.

### Age and Gender

**Figure 1:**
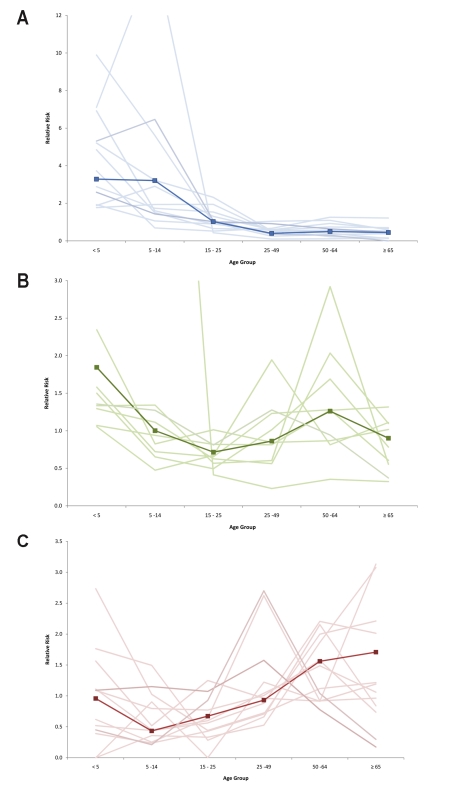
Relative risk of hospitalization, ICU admission, and death by age group compared to the general population. Countries included in hospitalization (A) and mortality (C) RRs: Japan, Hong Kong SAR, China, Singapore, Thailand, Chile, Germany, the Netherlands, Spain, New Zealand, Canada, US, Madagascar (hospitalizations only), and France (deaths only). Countries included in ICU admission RR (B): Japan, Hong Kong SAR, China, Singapore, Canada, Spain, the Netherlands, US, New Zealand, and South Africa. Dark line represents pooled RR; shaded lines are individual country RR.

**Figure 2:**
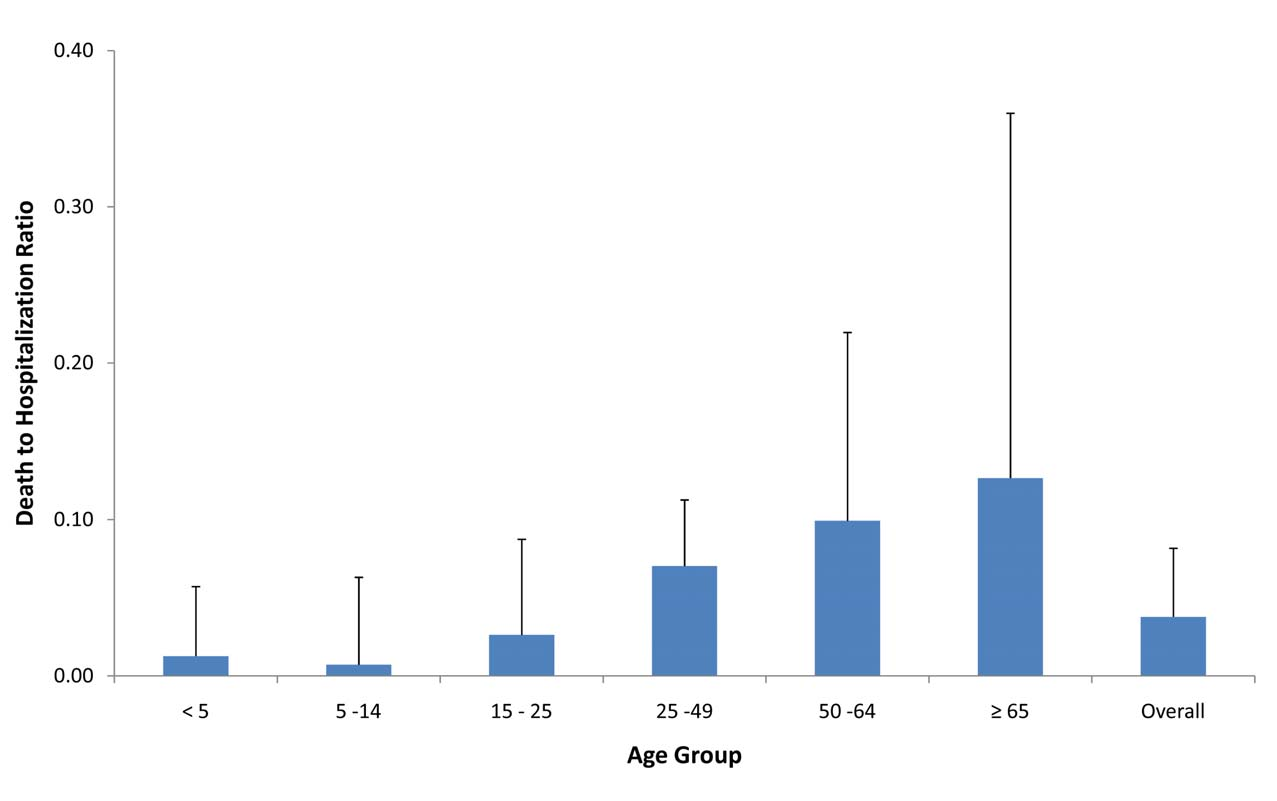
Ratio of confirmed H1N1pdm deaths to hospitalizations for selected countries. Countries included in figure: Spain, Singapore, China, Hong Kong SAR, Canada, the Netherlands, Thailand, Chile, Germany, Japan, US, and New Zealand. Bars represent maximum country ratio.

Approximately half of all patients included in this analysis in each level of severity were female (49.8%, 47.0%, and 44.7% of all hospitalized, ICU-admitted, and fatal H1N1pdm cases, respectively). This proportion did not vary significantly by country (Table 1). Age was associated with increased risk of poor outcome, as indicated by several different parameters. The median age of patients increased with increasing levels of severity (Table 1). Among hospitalized patients, the median age within each country ranged from 7 y in Japan to 38 y in Spain, with a median reported value among all countries that provided data (*n* = 14) of 19 y (IQR 14.8–27.5); among patients admitted to ICU, the median age within each country ranged from 28 y in China to 49.5 y in Hong Kong SAR, with a median value among all countries that provided data (*n* = 9) of 42 y (IQR 35.0–45.0); and among fatal cases, median age within each country ranged from 30 y in China to 56 y in Hong Kong SAR, with a median value among all countries that provided data (*n* = 13) of 46 y (IQR 37.0–42.0). When the age distribution of the proportion of patients in each level of severity was compared to the distribution in the general population, the RR was highest in the age groups <5 y and 5–14 y (RR_hosp_ = 3.3 and 3.2, respectively) but the RR of death was highest in the age groups 50–64 y and ≥65 y (RR_death_ = 1.6 and 1.7, respectively) (Figure 1). The ratio of H1N1pdm deaths to hospitalizations increased with age and was the highest in the ≥65-y-old age group in all countries for which data were available (Figure 2).

**Table 1:** Risk factors by severity level for select countries and risk of severe disease.

Risk Factor^a^	Severity Level^b^						RR of Severe Disease (IQR)^c^			
	*n* d	Hospitalized Cases	*n* d	ICU-Admitted Cases	*n* d	Fatal Cases	*n* d	RR_hosp_	*n* d	RR_death_
**Age**	14	19.0 (14.8–27.5)	9	42.0 (35.0–45.0)	13	46.0 (37.0–52.0)		—e		—e
**Gender (percent female)**	12	49.8 (46.2–51.5)	11	47.0 (41.9–50.5)	14	44.7 (41.5–48.7)	12	1.0 (0.8–1.1)	14	0.8 (0.7–1.0)
**Chronic medical illness**										
Respiratory disease	12	10.3 (5.0–21.7)	11	17.2 (10.5–29.9)	16	20.4 (9.3–29.5)	5	3.3 (2.0–5.8)	8	7.8 (4.9–26.6)
Asthma	11	17.6 (10.0–20.4)	9	9.8 (5.6–14.3)	15	5.3 (4.0–10.6)	3	1.8 (1.2–2.6)	6	1.7 (1.5–2.1)
Diabetes	14	9.0 (3.5–12.6)	12	13.6 (9.3–17.3)	17	14.4 (13.0–18.0)	7	0.9 (0.5–1.7)	10	4.0 (3.1–6.9)
Cardiac disease	12	7.1 (3.7–10.9)	11	10.9 (8.8–15.0)	15	12.1 (10.0–16.4)	6	2.0 (1.5–2.2)	8	9.2 (5.4–10.7)
Renal disease	13	4.0 (2.0–5.1)	11	6.3 (3.5–8.4)	16	7.1 (5.0–8.1)	2	4.4 (4.2–4.5)	3	22.7 (21.0–25.4)
Liver disease	9	1.1 (0.3–2.0)	9	2.4 (0.9–5.0)	12	4.9 (2.7–6.0)	3	5.7 (3.2–15.7)	4	17.4 (11.6–28.0)
Neurological disease	11	4.0 (2.5–7.5)	11	7.0 (3.5–9.5)	14	13.9 (5.5–18.4)	2	1.1 (0.9–1.3)	3	13.1 (8.4–32.4)
Immune compromised	13	5.0 (2.0–7.2)	11	6.7 (3.2–18.4)	15	12.5 (7.9–18.4)	2	24.3 (16.1–32.6)	4	27.7 (14.0–66.5)
Cases with ≥1 chronic medical illnesses	14	31.1 (19.0–47.1)	10	52.3 (41.1–58.7)	16	61.8 (48.5–67.9)		NA		NA
**Pregnancy** f										
First trimester	7	2.0 (1.0–3.5)	6	2.0 (1.5–2.5)	5	0.9 (0.0–2.5)				
Second trimester	7	7.0 (3.9–9.3)	7	5.0 (1.7–6.2)	5	2.5 (0.0–14.1)				
Third trimester	7	9.5 (7.6–21.3)	8	8.0 (4.0–14.6)	6	16.9 (5.1–32.0)				
Unknown trimester	8	6.0 (1.9–9.3)	6	2.8 (1.7–3.2)	7	0.0 (0.0–2.1)				
Total (any trimester)	10	17.4 (13.5–30.2)	9	15.0 (9.4–24.2)	11	6.9 (0.0–9.1)	10	6.8 (4.5–12.3)	11	1.9 (0.0–2.6)
**Obesity**										
BMI ≥30 or clinically obese	11	6.0 (1.5–7.5)	8	11.3 (7.9–15.8)	13	12.0 (10.0–21.0)	6	0.6 (0.2–1.8)	7	1.5 (0.9–2.8)
BMI = 30–40	3	7.0 (4.4–16.0)	3	10.0 (6.9–18.5)	4	15.8 (7.7–25.2)		NA		NA
BMI >40	5	3.0 (1.4–11.5)	5	5.0 (3.4–16.4)	6	15.2 (4.0–30.8)	2	15.0 (9.5–20.4)	2	36.3 (22.4–50.1)
BMI not measured but judged clinically obese	8	4.3 (1.8–13.3)	4	4.4 (3.4–5.3)	8	7.8 (3.8–17.3)		NA		NA
**Vulnerable social/ethnic group**	4	5.2 (2.3–10.6)	4	5.0 (1.5–10.7)	4	10.1 (5.3–18.5)	4	1.0 (0.2–3.7)	4	2.4 (1.2–3.8)
**TB**	2	1.7 (0.9–1.8)	2	1.3 (1.0–1.6)	4	2.6 (0.8–5.9)		NA		NA

a See Text S1 for definitions of risk factors.

b All data given as median percent (IQR), except for age, which is median (in years) (IQR).

c RR_hosp_ is the unadjusted RR of hospitalization among H1N1pdm patients with the risk factor compared to the risk of hospitalization among H1N1pdm patients without the risk factor, and RR_death_ is the unadjusted RR of death among H1N1pdm patients with the risk factor compared to the risk of death among H1N1pdm patients without the risk factor; range of RR provided if ≥2 countries provided data.

d The number of countries providing data for cell directly to the right; the full list of countries that provided data for each risk factor is provided in Text S1.

e RR_hosp_ and RR_death_ calculated by age group and shown in Figure 1.

f Denominator is women of childbearing age in each level of severity.

NA, not assessed.

### Chronic Illness

**Figure 3:**
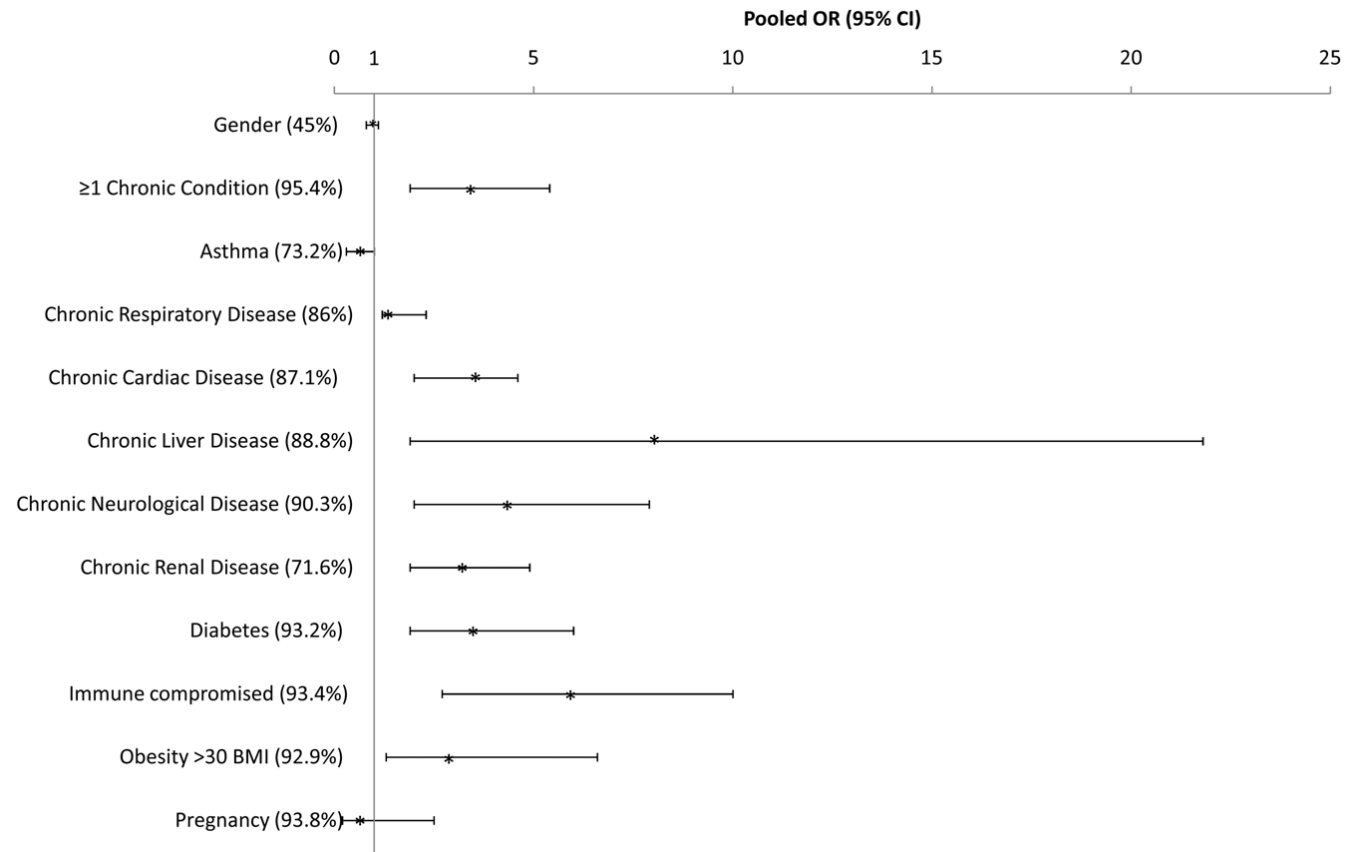
Pooled odds ratio and 95% CIs of risk of death given hospitalization for selected countries. See Text S1 for countries included in the pooled risk factor ORs.

The proportion of H1N1pdm patients with at least one chronic medical condition generally increased with severity (median among all countries that provided data was 31.1% [*n* = 14], 52.3% [*n* = 10], and 61.8% [*n* = 16] of hospitalized, ICU-admitted, and fatal H1N1pdm cases, respectively (Table 1). This pattern was observed for most countries (individual country data not shown). For nearly every individual risk factor under study, the prevalence increased significantly with severity level. Chronic respiratory conditions excluding asthma (median = 10.3%, 17.2%, and 20.4%, respectively) and asthma (median = 17.6%, 9.8%, and 5.3%, respectively) were the risk factors most often reported among severe cases, followed closely by diabetes (median = 9.0%, 13.6%, and 14.4%, respectively) and chronic cardiac conditions (median = 7.1%, 10.9%, and 12.1%, respectively). The pooled OR for death given hospitalization was significantly above one for each risk factor listed, with the exception of asthma, and was highest for chronic liver disease and immunocompromised patients (Figure 3).

The risk of severe disease due to H1N1 infection, including hospitalization and death, was elevated for every chronic condition for which data were available (Table 1). Notably, the RR for fatal disease due to H1N1pdm infection was elevated for asthma (median RR_death_ = 1.7 [IQR 1.5–2.1]) and not markedly different from the RR associated with hospitalizations (median RR_hosp_ = 1.8 [IQR 1.2–2.6]). Data on chronic illness rates in the general population were not available from enough countries to permit an assessment of the relative magnitude of risk associated with various conditions with certainty.

### Pregnancy

The proportions of women of childbearing age who were hospitalized with H1N1 and were pregnant as part of all hospitalizations (median of all country data = 17.4% [IQR 13.5–30.2]), who were admitted to ICU (median of all country data = 15.0% [IQR 9.4–24.2]), and who died (median of all country data = 6.9% [0.0–9.1]) varied within each country. Pregnant women in their third trimester consistently accounted for more than half of all pregnant women among hospitalized, ICU-admitted, and fatal cases. However, with the exception of China, Thailand, and the US, the proportion of pregnant women decreased with increasing level of severity, and the pooled OR for death given hospitalization during pregnancy was below 1 (pooled OR = 0.6, 95% CI 0.2–2.5).

Pregnant women with H1N1pdm infection were at higher risk of hospitalization than women of childbearing age in the general population without H1N1pdm infection, with an unadjusted RR of hospitalization ranging from 3.5 in Germany to 25.3 in France (median RR_hosp_ = 6.8, *n* = 10 countries). The unadjusted RR of death, while elevated compared to non-pregnant women in more than half of countries, was generally lower than that for hospitalization, with a median RR_death_ of 1.9 (*n* = 11 countries). Four areas (Japan, the Netherlands, Hong Kong SAR, and Singapore) had a RR_death_ of zero.

### Other Risk Factors

The proportion of patients with obesity (body mass index [BMI] ≥30 or clinically judged as obese) increased with increasing disease severity and represented a median of 6%, 11.3%, and 12.0% of all hospitalized, ICU-admitted, and fatal H1N1pdm cases, respectively, and this pattern was also observed for morbid obesity (BMI >40), with 3.0%, 5.0%, and 15.2%, respectively (Table 1). However, this pattern was not consistently reported in each country. For example, France, Thailand, and China observed similar proportions of obese patients among ICU-admitted and fatal cases, while Hong Kong SAR reported a lower prevalence of obesity among fatal cases than among ICU admissions. Using data from all countries, the pooled OR for death given hospitalization for obesity (BMI ≥30 or clinically judged as obese) was 2.9 (95% CI 1.3–6.6; Figure 3). Compared to the general population in the two countries for which data were available, the risk of death associated with morbid obesity was increased (mean RR_death_ = 36.3 [IQR 22.4–50.1], *n* = 2).

Canada, Australia, and New Zealand reported significant disparities in the burden of severe H1N1pdm disease across different ethnic groups. In these three countries, indigenous population groups were overrepresented among severe H1N1pdm cases requiring hospitalization and among fatal cases. In contrast, in Thailand and Mexico, minority groups were underrepresented among severe H1N1pdm cases. Taken together, the unadjusted median RR of hospitalization for H1N1pdm patients among minority groups was 1.0 (IQR 0.2–3.7) and the median RR of death was 2.4 (IQR 1.2–3.8). TB data were reported from three countries, and the incidence increased slightly with level of severity. The disease was reported in a median of 1.7%, 1.3%, and 2.6% of hospitalized, ICU-admitted, and fatal H1N1pdm cases, respectively. We were not specifically able to evaluate HIV incidence because of a paucity of data on HIV in H1N1pdm patients.

## Discussion

Our analysis represents to our knowledge the first comprehensive assessment of the frequency and distribution of risk factors for severe H1N1pdm infection from a global perspective, with data from approximately 70,000 patients requiring hospitalization, 9,700 patients admitted to ICU, and 2,500 fatalities from 19 countries and administrative regions around the world. Consistent with other published data, our results reaffirm that the age distribution of severe H1N1pdm cases significantly differs from that of seasonal influenza [53]–[56]. The highest rates of hospitalization per capita were in children <15 y, but the highest rates of mortality per capita were in persons over 64 y. The low apparent attack rate in the oldest age group, evidenced by low rates of hospitalization, and the high odds associated with age in the fatal group compared to hospitalized cases seems to indicate that although older adults may have a lower risk of infection, they have a significantly higher risk of death if they are infected [54],[57]–[61]. It is likely that increasing prevalence of chronic risk conditions in the oldest age group contributes to this effect, but our data do not allow for quantification of this association.

Our results demonstrate that in a significant portion of severe and fatal cases, patients had preexisting chronic illness, and that the presence of chronic illness increased the likelihood of death. It was notable, however, that approximately 2/3 of hospitalized cases and 40% of fatal cases did not have any identified preexisting chronic illness. It is unknown how many of these cases had other risk factors, such as pregnancy, obesity, and substance abuse (including smoking and alcohol), for which we had insufficient information in this study. These figures are also dependent on the completeness of available data for recorded risk factors. As with seasonal influenza, the most common underlying chronic conditions among hospitalized patients were respiratory disease, asthma, cardiac disease, and diabetes. Interestingly, we found that although asthma was frequently associated with both hospitalization and death in most countries, with an increased RR for both, the OR for death given hospitalization suggested that a higher proportion of hospitalized cases survived compared to patients with other conditions. This may represent the occurrence of manageable influenza-induced exacerbations of asthma prompting admission that do not progress to viral pneumonia or other fatal complications, and may also reflect the fact that asthma tends to occur in younger age groups [62].

Early data suggested that pregnancy might be an important risk factor for severe disease with H1N1pdm [21],[25],[63],[64]. Our analysis is consistent with these reports and more recent studies [47],[65], which found an overall trend that pregnant women, mainly in their third trimester, have a higher incidence of hospitalization than the general population. Several published studies have also shown that pregnancy is associated with a higher risk of ICU admission and fatal outcome [54],[58],[66],[67]. In our analysis, the risk associated with pregnancy was elevated for both hospitalization and fatality compared to women of childbearing age, though the latter association was not consistently observed in every country. As with asthma, the proportion of pregnant women generally decreased with severity level for most of the countries. Our results suggest that pregnant women with H1N1pdm are approximately seven times more likely to be hospitalized and two times more likely to die than non-pregnant women with H1N1pdm. The greater risk for hospitalization than for death with H1N1pdm influenza infection during pregnancy may have resulted from a lower threshold for admitting infected pregnant women to hospital and/or a more aggressive approach to antiviral or other treatment for pregnant women. In addition, the occurrence of non-respiratory complications of pregnancy, such as hypertension, pre-eclampsia, and premature labor, provoked by H1N1pdm infection may have increased the risk of hospitalization while not resulting in death [68]. This would be consistent with published reports of case series of pregnant patients that list complications of pregnancy as a common cause of admission [63],[69],[70]. The dataset did not allow us to adjust for underlying conditions in pregnant women, and thus to distinguish between risks for healthy pregnant women, and pregnant women with underlying medical conditions; however, we believe that the results support an approach of early intervention with pregnant women who develop influenza.

Early in the 2009 pandemic, clinicians from the US reported a surprisingly high prevalence of morbid obesity, a risk factor not previously associated with severe outcomes for seasonal influenza infection, in patients with severe complications of H1N1pdm infection [71]. Subsequent studies in several countries, including the US, Mexico, Canada, Spain, Greece, France, Australia, and New Zealand, reported high proportions of obesity among ICU admissions and fatal cases [13],[20],[58],[64],[72]–[77]. Our results provide supportive evidence that obesity may be a risk factor for severe disease, as seen in the increasing proportion of morbidly obese patients with severity level and the associated elevated OR. Our findings also suggest that morbidly obese patients with H1N1pdm are more likely to die if hospitalized; however, the results in our analysis were not consistent across all countries. The association between obesity (or morbid obesity) and severe outcomes may reflect direct causation (e.g., due to greater respiratory strain of infection on obese individuals), causation through other known risk factors (e.g., obesity causes diabetes and heart disease, which pose an increased risk for severe outcome [36]), or a noncausal association, if some other factor (e.g., genetic or dietary) caused both morbid obesity and increased risk of severe outcome. Unfortunately, our dataset did not allow us to distinguish among these nonexclusive alternatives.

Indigenous populations and ethnic minorities have been reported to experience a disproportionately high burden of severe H1N1pdm infection, particularly in the Americas [14],[21],[23],[36],[64],[75],[78]–[80] and the Australasia-Pacific region [43],[80]–[82], similar to reports during the 1918 influenza pandemic [83]–[85]. Our analysis of Australian, New Zealand, and Canadian data concur with these published reports, and while compelling, were not universal. Neither Thailand nor Mexico observed a significantly increased burden of severe H1N1pdm disease among indigenous or minority populations. Our data are not sufficient to explain the observed differences in the reported risk of severe disease among minority groups, but several hypotheses have been proposed, including a higher prevalence of chronic medical conditions known to increase risk of severe influenza, delayed or reduced access to healthcare, cultural differences in healthcare-seeking behavior and approaches to health, potential differences in genetic susceptibility, and social inequalities [23],[78],[80]. More research is needed to better understand and quantify the increased risk of severe H1N1pdm disease among these groups. However, an imperfect understanding of the mechanisms of health disparities related to severe H1N1pdm disease should not impede the public health community in undertaking actions to mitigate this risk by disseminating appropriate public information, targeting outreach and prevention programs, and involving at-risk population groups in pandemic planning.

Our analysis has a number of limitations, not least of which is the wide differences in surveillance systems, case management policies, and antiviral use in the countries studied. The criteria and indications for hospital and ICU admission for certain conditions (e.g., pregnancy and asthma) and by age (e.g., pediatric patients) varied significantly by country, and may have been somewhat dependent on capacity for admission, which likely varied over time. Risk factors are also dependent on the completeness and quality of data on risk factors reported and classification of death in the absence of complete testing. These variables could lead to a bias in the estimate of these conditions among severe cases and could make direct comparisons across countries difficult. Second, our data do not consider multiple risk factors for individual H1N1pdm patients. A lack of individual-level data on underlying medical conditions of H1N1pdm patients precludes our ability to sufficiently control for confounding and therefore identify the independent contribution of individual risk factors for severe disease and death. The differences observed in risk factors for hospitalization and death among H1N1pdm patients compared to among seasonal influenza patients, and the wide range of RR values between countries may be explained by differences in age structure in the general population. Several studies have identified important differences in the proportions of underlying conditions by age among hospitalized and fatal cases, including, but not limited to, the UK [15],[53], the US [36], Canada [47], and Singapore [39],[86].

A third limitation is related to our imperfect calculation of the point prevalence of pregnancy among women of childbearing age in the general population. However, we believe that our findings of the range of RR values for hospitalization and death is valid, but may be very slightly inflated because of undercounting in the denominator. The inflationary effect of undercounting is likely greatest for pregnant women in the first trimester, as we didn't adjust for common first trimester events such as miscarriages or abortions, and in this group there is likely substantial undercounting in the numerator as well because of women not knowing they are pregnant in that period. Fourth, the data used in our analysis relied on hospital records, which were not standardized, and were likely to be incomplete or vary in quality between hospitals or countries. This poses a problem in the direct comparativeness between settings.

Despite these limitations, this analysis is the first to our knowledge to compare risk factors across a variety of countries using data from a very large number of patients, and we found a great deal of consistency for much of the data. Clearly, cardiac disease, chronic respiratory disease, and diabetes are important risk factors for severe disease that will be especially relevant for countries with high rates of these illnesses. We provide evidence to support the concern regarding obesity, particularly morbid obesity, as a risk factor, though this needs more study. We found large between-country variations for some important risk factors, most notably pregnancy, and the reasons for these differences need more study. There is evidence to suggest that the differences observed for pregnancy might represent differences in case management practices, and we believe that the available evidence supports vaccination and early intervention for pregnant women. Our study reinforces the need to identify and target high-risk groups for interventions, such as immunization, information, early medical advice, and use of antiviral medications. Experience with the 2009 H1N1 pandemic and the differences observed between countries have highlighted the need for country-specific surveillance data and global standardization of case definitions and data collection, and the usefulness of data sharing to aid policy makers in critical decision making for global influenza epidemics.

## Supporting Information

Text S1 **Supplemental data and analysis.**
(PDF)

## Abbreviations

BMI: body mass index
CI: confidence interval
ICU: intensive care unit
IQR: interquartile range
OR: odds ratio
RR: relative risk
TB: tuberculosis
WHO: World Health Organization
