# Supplementary material for: Risk Factors for Severe Outcomes following 2009 Influenza A (H1N1) Infection: A Global Pooled Analysis
Source: PLoS Med. 2011 Jul 5;8(7):e1001053. doi: 10.1371/journal.pmed.1001053 (PMC3130021; doi:10.1371/journal.pmed.1001053)
Supplement: Text S1 — Supplemental data and analysis. (PDF) [file pmed.1001053.s001.pdf]

Supplemental Information:

Risk factors for severe outcomes following 2009 Influenza A (H1N1) infection: A Global Pooled Analysis

WHO Working Group for Risk Factors for Severe H1N1pdm Infection†

†Maria D Van Kerkhove[1,2], Katelijn AH Vandemaele[1], Vivek Shinde[1], Giovanna Jaramillo-Gutierrez[1], Artemis Koukounari[2], Christl Donnelly[2], Luis O. Carlino[3], Rhonda Owen[4], Beverly Paterson[4], Louise Pelletier[5], Julie Vachon[5], Claudia Gonzalez[6], Yu Hongjie[7], Feng Zijian[7], Shuk Kwan Chuang[8], Albert Au[8], Silke Buda[9], Gerard Krause[9], Walter Haas[9], Isabelle Bonmarin[10], Kiyosu Taniguichi[11], Kensuke Nakajima[12], Tokuaki Shobayashi[12], Yoshihiro Takayama[12], Tomi Sunagawa[11]; Jean Michel Heraud[13], Arnaud Orelle[13], Ethel Palacios[14], Marianne AB van der Sande[15], CCH Lieke Wielders[15], Darren Hunt[16], Jeffrey Cutter[17], Vernon Lee[18, 19], Juno Thomas[20], Patricia Santa-Olalla[21], Maria J. Sierra-Moros[21], Wanna Hanshaoworakul[22], Kumnuan Ungchusak[22], Richard Pebody[23], Seema Jain[24], Anthony W Mounts[1*]

1 Global Influenza Programme, World Health Organization
2 MRC Centre for Outbreak Analysis and Modelling, Department of Infectious Disease Epidemiology, Imperial College London, London UK
3 Ministerio de Salud de la Nación, Buenos Aires, Argentina
4 Influenza Surveillance Section, Surveillance Branch, Office of Health Protection, Department of Health and Ageing, Australia
5 Influenza Surveillance Section, Public Health Agency of Canada, Ontario, Canada
6 Departamento de Epidemiología, División de Planificación Sanitaria, Ministerio de Salud de Chile
7 Office for Disease Control and Emergency Response, Chinese Center for Disease Control and Prevention Beijing, P.R.China
8 Surveillance and Epidemiology Branch, Centre for Health Protection, Centre for Health Protection of Department of Health, Hong Kong SAR
9 Department for Infectious Disease Epidemiology, Robert Koch Institute, Berlin, Germany
10 Département des maladies infectieuses, Institut de Veille, Sanitaire, Saint-Maurice Cedex, France
11 Infectious Disease Surveillance Center, National Institute of Infectious Diseases, Tokyo, Japan
12 Ministry of Health, Labour and Welfare Japan
13 Virology Unit, Institut Pasteur from Madagascar, Antananarivo, Madagascar
14 Directorate General of Epidemiology, FCO, De P. Miranda, Mexico City, Mexico
15 Epidemiology and Surveillance Unit, Centre for Infectious Disease Control, National Institute for Public Health and the Environment, Bilthoven, the Netherlands
16 New Zealand Ministry of Health, Wellington, New Zealand
17 Communicable Diseases Division at the Ministry of Health, Singapore
18 Biodefence Centre, Ministry of Defence, Singapore
19 Department of Epidemiology and Public Health, Yong Loo Lin School of Medicine, National University of Singapore, Singapore
20 Epidemiology and Surveillance Unit, Respiratory Virus Unit, National Institute for Communicable Diseases, a division of the National Health Laboratory Service, Johannesburg, South Africa
21 Coordinating Centre for Health Alerts and Emergencies, Dirección General de Salud Pública y Sanidad Exterior Ministerio de Sanidad y Política Social, Spain
22 Department of Disease Control, Ministry of Public Health, Nonthaburi, Thailand
23 Health Protection Agency, England
24 Epidemiology and Prevention Branch, Influenza Division, Centers for Disease Control and Prevention, Atlanta, GA

1 Standardized reporting of underlying risk conditions in human cases of pandemic (H1N1) 2009

1.1 Background, rational, and application of standardized reporting of underlying risk conditions

Since the start of the 2009 H1N1 pandemic, several countries have published estimates of the proportion of pandemic H1N1 2009 cases with an underlying risk condition and have described high risk groups. However, compilation of data internationally, making comparisons between countries, and monitoring for changes over time have been difficult due to a lack of a standardized approach to data gathering and reporting of risk conditions. In collaboration with several partners, WHO has developed a recommended standardized format for reporting underlying risk conditions associated with cases of pandemic H1N1 2009.

Tracking the proportion of severe and fatal pandemic H1N1 2009 cases with underlying risk conditions over time is a critical part of monitoring the evolution of the pandemic, monitoring for changes in the epidemiology of the disease, and assessing changes in the severity of virus infection. A clear understanding of risk groups will allow policy makers to adapt recommendations regarding vaccination, antiviral use, and non pharmaceutical control strategies.

Standardization of reporting will facilitate:

1. Comparability of data across time and geographical areas to facilitate monitoring of risk groups for changes in attack patterns or virulence of pandemic influenza.
2. Development and refinement of targeted intervention strategies for groups at increased risk for poor outcomes.
3. Standardization of language for communication purposes.

1.2 Risk factors for severe outcomes with influenza infections

Several conditions that increase the risk of severe outcomes from influenza have been described for seasonal influenza [1,2,3]. Early epidemiological data from the current pandemic suggest that these previously recognized underlying risk conditions for seasonal influenza may be similar for pandemic H1N1 2009 influenza. These risk factors can be grouped into three categories.

1. Chronic medical illnesses such as chronic lung disease and diabetes
2. Pregnancy
3. Extremes of age

As new data on the pandemic emerges, other conditions, such as obesity have been proposed as risk factors for severe disease and members of some indigenous populations have been noted to have higher rates of hospitalization and death. At the time of writing of this guidance, these factors have not yet been definitively shown to increase risk of severe outcomes independently

of other previously accepted risk factors and so should be reported as a separate category. The separate reporting of these factors will greatly facilitate achieving a better understanding of their role in increasing the risk of poor outcome with pandemic influenza.

Finally, due to the high prevalence in some parts of the world of certain medical conditions for which the risk in relation to influenza is less well understood, (for example, tuberculosis and malnutrition) there is a need to quantify the risk associated with these conditions.

Currently, reporting of risk factor data is sometimes confused by the inclusion of other chronic medical conditions that are commonly diagnosed but that have not been associated with severe outcomes such as hypertension in the absence of associated heart disease, smoking in the absence of associated lung disease, and hyperlipidemia in the absence of associated cardiovascular disease. Inclusion of these conditions in the reporting of underlying comorbid conditions complicates the interpretation of risk factor data.

The recommended standardized reporting format for underlying risk conditions is not intended to be an additional formal reporting requirement, but rather, an effort to provide guidance on standardized reporting of underlying risk conditions where that information exists or is already being collected and reported. WHO encourages countries that collect these data to report them using this format.

1.3 Recommended reporting format for recognized underlying risk conditions

In order to facilitate WHO recommends the separate reporting of the categories of risk conditions as outlined above (chronic medical illness, pregnancy, age, and other) for different levels of poor outcome. Where data are available, the proportions with recognized underlying risk conditions among confirmed H1N1 cases should be reported for three severe outcome groups: those hospitalized, those requiring intensive care or mechanical ventilation, and those who die (Table S1).

Therefore, risk conditions associated with these severe outcomes should be categorized as follows:

- Proportion of patients in each of the three severe-outcome groups with at least one recognized underlying high-risk chronic medical illness.
- Proportion of patients in each of those poor outcome groups that are pregnant.
- Proportion of patients in each of the three severe outcome groups by age: <5 years old, 5 to <15 years old, 15 to < 25 years old, 25 to <50 years old, 50 to <65 years old, and ≥65 years old.
- Proportion in each of the severe outcome groups with other putative risk factors such as obesity, membership in an indigenous population or other suspect high-risk group, tuberculosis, malnutrition, or other groups suspected to be at high-risk by national health authorities.

The last group will differ by country and depend on the local interest of public health authorities. Help for definition on some conditions, such as malnutrition, can be found in other WHO publications.

It is expected that member states with adequate resources for doing detailed data collection and analysis will gather additional detailed data on risk factors and this is encouraged. The recommendations included in this annex and the tables that follow are meant to be used for summary reporting of risk factor data to WHO and internationally in a standard format.

Table S1: Suggested classification scheme for Influenza H1N1 risk factor data reporting*

	**Hospitalizations**	**Intensive**	**Deaths**
	**n (% of all H1N1 hospitalizations)**	**n (% of all H1N1 intensive care admissions)**	**n (% of all H1N1 deaths)**
**Gender (% female)**			
**≥ 1underlying chronic medical illness listed below preceding H1N1 infection**			
Chronic respiratory disease			
Asthma			
Diabetes			
Chronic cardiac disease			
Chronic renal disease			
Chronic liver disease			
Chronic neurological disease			
Immune compromise			
**Pregnancy**			
First trimester (1-12 wks)			
Second trimester (13-27 wks)			
Third trimester (28-40+ wks)			
**Total:**			
**Median age**			
**Age groups**			
< 5 yr old			
5 -14 yr old			
15 - 25 yr old			
25 -49 yr old			
50 -64 yr old			
≥ 65 yr old			
**Other conditions**			
Obesity			
Body Mass Index 30 - 40			
Body Mass Index > 40			
Vulnerable social/ethnic group			
Tuberculosis			

* See table S2 below for risk factor definitions and explanations

Table S2. Risk Condition Definitions

Risk Condition	Examples, definitions:
**Death associated with pandemic influenza (H1N1) 2009**	• With laboratory confirmed pandemic (H1N1) virus infection, either ante- or post-mortem<br>• Regardless of another primary or contributing cause of death<br>• Without any period of complete recovery between onset of influenza-related illness and death
**Laboratory confirmed case**	An individual with laboratory-confirmed pandemic (H1N1) 2009 virus infection by one or more of the following tests:<br>• polymerase chain reaction (PCR);<br>• viral culture;<br>• 4-fold rise in pandemic (H1N1) 2009 virus virus-specific neutralizing antibodies.
**Chronic respiratory disease**	• Chronic obstructive pulmonary disease (COPD) including chronic bronchitis and emphysema; bronchiectasis, cystic fibrosis, interstitial lung fibrosis, pneumoconiosis and bronchopulmonary dysplasia (BPD)
**Asthma**	• For example, asthma that requires continuous or repeated use of bronchodilators, inhaled or systemic corticosteroids, or with previous exacerbation requiring hospital admission.
**Diabetes mellitus**	• Type 1 diabetes<br>• Type 2 diabetes requiring insulin or oral hypoglycemic drugs
**Chronic cardiac disease**	Conditions that require regular medications and/or follow up including:<br>• Congenital heart disease<br>• Cardiomyopathy as the result of prolonged hypertension (hypertension alone in the absence of associated heart disease is not considered a risk factor for severe outcome)<br>• Chronic heart failure<br>• Individuals requiring regular medication and/or follow-up for ischaemic heart disease
**Chronic renal disease**	• Chronic renal failure<br>• Nephrotic syndrome<br>• Renal transplantation
**Chronic liver disease**	• Cirrhosis<br>• Biliary atresia<br>• Chronic hepatitis
**Chronic neurological disease**	• Stroke<br>• Neuromuscular diseases that lead to impaired respiratory function or aspiration risk such as cerebral palsy or myasthenia gravis
**Immune compromise (through disease or treatment)**	• Immunodeficiencies related to use of immunosuppressive drugs (e.g. chemotherapy) or systemic steroids<br>• Asplenia or splenic dysfunction (sickle cell anemia)<br>• Human Immunodeficiency Virus infection and Acquired Immune Deficiency Syndrome.
**Obesity parameter, Body Mass Index (BMI)**	BMI is calculated as body weight in kilograms divided by the square of the height in meters (kg/m^2). WHO defines obesity as a BMI of > 30 kg/m^2. A commonly used definition for extreme or morbid obesity is a BMI > 40 kg/m^2
**Socially defined risk groups**	Indigenous populations (self identified), racial and ethnic minorities, other identifiable groups with limited access to health care or high prevalence of chronic illness.
**Tuberculosis**	History of previous or current *symptomatic* tuberculosis requiring treatment.

2 Available data by risk factor

Descriptions of influenza surveillance systems in individual countries, which were used to provide data on risk factors among H1N1pdm confirmed patients are available from the following sources [4,5,6,7,8,9,10,11,12,13,14,15,16,17,18,19,20,21,22,23,24,25,26,27].

Data on chronic conditions and all other risk factors among hospitalized, ICU and fatal H1N1pdm patients available by country are provided in Table S3, Table S4 and Table S5, respectively.

Obesity among H1N1pdm patients was categorized in four groups: 1) patients with body mass index (BMI) between 30 and 40, 2) BMI >40 (morbidly obese), 3) patients for which no BMI was measured but who were clinically judged to be obese, 4) BMI ≥30 or judged to be clinically obese.

Data on the proportions of vulnerable social or ethnic groups represented among hospitalized ad fatal H1N1pdm patients were available from Canada, Thailand, Mexico and New Zealand (fatal patients only). Data on the proportions of vulnerable social or ethnic groups represented among ICU patients was available from Canada and Thailand. Data on the proportions of persons identified as aboriginal in Australia among hospitalized, ICU-admitted, and fatal cases were abstracted from a weekly influenza surveillance report published by the Australia Department of Aging [28].

Table S3 Countries which contributed data on hospitalized H1N1 laboratory confirmed cases in association with individual characteristics or risk factors

	Risk Factor																
															Obesity		
Country	Age	Gender	≥1 Chronic Condition	Chronic respiratory disease	Asthma	Diabetes	Chronic cardiac disease	Chronic renal disease	Chronic liver disease	Chronic neurological disease	Immune compromised	Vulnerable social/ ethnic group	TB	Pregnancy	BMI 30-40	BMI >40	BMI not measured but judged clinically obese
Argentina	x																
Australia				x		x	x	x		x	x	x					
Canada	x	x	x	x	x	x	x	x	x	x	x	x		x			
Chile	x		x	x	x	x	x	x	x	x	x						x
China	x	x	x	x	x	x	x	x	x	x	x		x	x	x	x	
France	x		x	x	x	x	x	x		x	x			x		x	
Germany	x	x	x			x					x			x			x
Hong Kong SAR	x	x	x	x		x	x	x	x		x			x			
Japan	x	x	x			x	x	x	x	x	x			x			
Madagascar	x	x	x		x												
Mexico		x	x	x	x	x		x				x					x
the Netherlands	x	x	x	x	x	x	x	x	x	x	x			x			x
New Zealand																	
Singapore	x	x	x	x	x	x	x	x	x	x	x			x			x
South Africa																	x
Spain	x	x	x	x	x	x	x	x	x	x	x			x		x	
Thailand	x	x	x	x	x	x	x	x	x	x	x	x	x		x	x	x
United States	x	x	x	x	x	x	x	x		x	x			x	x	x	x
United Kingdom																	

Note: "x" indicates data was contributed by named country

Table S4 Countries which contributed data on H1N1 laboratory confirmed cases admitted to ICU in association with individual characteristics or risk factors

	Risk Factor																
														Obesity			
Country	Age	Gender	≥1 Chronic Condition	Chronic respiratory disease	Asthma	Diabetes	Chronic cardiac disease	Chronic renal disease	Chronic liver disease	Chronic neurological disease	Immune compromised	Vulnerable social/ethnic group	TB	Pregnancy	BMI 30-40	BMI >40	BMI not measured but judged clinically obese
Argentina																	
Australia				x		x	x	x		x	x	x					
Canada	x	x	x	x	x	x	x	x	x	x	x	x		x			x
Chile																	
China	x	x	x	x	x	x	x	x	x	x	x		x	x	x	x	
France	x	x	x	x	x	x	x	x	x	x	x			x		x	
Germany																	
Hong Kong SAR	x	x	x	x	x	x	x	x	x	x	x			x			
Japan		x	x			x	x	x	x	x	x			x			
Madagascar		x															
Mexico												x					
the Netherlands	x	x	x	x	x	x	x	x	x	x	x			x			x
New Zealand				x		x											
Singapore	x	x	x	x	x	x	x	x	x	x	x			x			x
South Africa																	
Spain	x	x	x	x	x	x	x	x	x	x	x			x		x	
Thailand	x	x	x	x	x	x	x	x	x	x	x	x	x		x	x	x
United States	x	x	x	x	x	x	x	x		x	x			x	x	x	
United Kingdom																	

Note: "x" indicates data was contributed by named country

Table S5 Countries which contributed data on fatal H1N1 laboratory confirmed cases in association with individual characteristics or risk factors

	Risk Factor																
														Obesity			
Country	Age	Gender	≥1 Chronic Condition	Chronic respiratory disease	Asthma	Diabetes	Chronic cardiac disease	Chronic renal disease	Chronic liver disease	Chronic neurological disease	Immune compromised	Vulnerable social/ ethnic group	TB	Pregnancy	BMI 30-40	BMI >40	BMI not measured but judged clinically obese
Argentina																	
Australia				x		x	x	x		x	x	x					
Canada	x	x	x	x	x	x	x	x	x	x	x	x		x			
Chile	x		x	x	x	x	x	x	x	x	x						x
China	x	x	x	x	x	x	x	x	x	x	x		x	x	x	x	
France	x	x	x	x	x	x	x	x	x	x	x			x		x	
Germany		x	x			x					x			x			x
Hong Kong SAR	x	x	x	x	x	x	x	x	x	x	x			x			
Japan	x	x	x	x	x	x	x	x	x	x	x		x	x			
Madagascar		x															
Mexico		x	x	x	x	x		x									x
the Netherlands	x	x	x	x	x	x	x	x	x	x	x			x		x	x
New Zealand	x		x	x	x	x	x		x	x	x	x					
Singapore	x	x	x	x	x	x	x	x	x	x	x			x			x
South Africa	x		x	x	x	x	x	x					x				x
Spain	x	x	x	x	x	x	x	x	x	x	x			x		x	
Thailand	x	x	x	x	x	x	x	x	x	x	x	x	x		x	x	x
United States	x	x	x	x	x	x	x	x		x	x			x	x	x	x
United Kingdom		x	x	x	x	x	x	x	x	x	x			x			

Note: "x" indicates data was contributed by named country

3 Calculation of Relative Risk of Hospitalization and Death

Data on the prevalence of individual risk factors in the general population were provided from published sources from Germany[29], the Netherlands [30,31], US (NHANES 2007-2008), Singapore [16], Spain [32,33,34], France [35], New Zealand (Health and Disability Intelligence Unit, Ministry of Health, unpublished data), Japan [36,37], Canada[38], Hong Kong SAR [39,40,41,42], and China [43,44,45,46,47,48,49,50,51,52,53,54,55,56,57]. 2010 estimates of population by age was obtained from the United Nations[58]. Using data provided by countries, we calculated the relative risk of hospitalization (RRhosp) as:

RR Hospitalization =

$$\frac{\% \, hospitalized \, patients \, with \, riskfactor}{\% \, in \, general \, population \, with \, risk \, factor} \Bigg/ \frac{\% \, of \, hospitalizated \, patients \, without \, riskfactor}{\% \, in \, general \, population \, without \, risk \, factor}$$

and relative risk of death (RRdeath) as:

RR death =

$$\frac{\% \, fatal \, patients \, with \, riskfactor}{\% \, in \, general \, population \, with \, risk \, factor} \Bigg/ \frac{\% \, of \, fatal \, patients \, without \, riskfactor}{\% \, in \, general \, population \, without \, risk \, factor}$$

Countries included in the calculation of RR hospitalization and RR death for age (Figure 1a and b, Main text) include Japan, Hong Kong, China, Singapore, Thailand, Chile, Germany, the Netherlands, Spain, Canada, US, France (deaths only). Countries included in the calculation for RR by age of ICU (Figure 1c, Main Text) include Japan, Hong Kong, China, Singapore, Canada, Spain, the Netherlands, USA, South Africa.

4 Pooled Odds Ratio Calculations

Country-specific odds ratios (OR) and 95% confidence intervals (CI) for death were calculated separately for each risk factor (i.e., the odds of death given hospitalization and a specific risk factor), thereby comparing the odds of death in one group (for example, among hospitalized patients with asthma) with the odds of death in all others combined (for example, among hospitalized patients without asthma) (individual country ORs not shown). We then used the I^2 statistic to quantify the percentage of variation across countries that is due to true underlying heterogeneity in the OR rather than chance variability [59]. The I^2 statistics for all examined risk factors indicated that there was substantial true underlying variation between ORs from different countries. Thus, we undertook a random effects meta-analysis to describe the distribution of the OR estimates across the countries for which data were available for analysis. Underlying this approach is the assumption that, although the individual countries give rise to different OR estimates, these estimates arise from a distribution with a central value, the estimate of which is referred to as the “pooled OR”, and normally distributed variability around this value.

Countries with ≥25 confirmed hospitalized or fatal cases were included in the analysis. Countries included in gender pooled OR: Hong Kong, China, Thailand, Singapore, Canada, Madagascar,

Spain, the Netherlands, Germany, Japan and US; Countries included in ≥1 condition pooled OR: Hong Kong, China, Thailand, Canada, Madagascar, the Netherlands, Germany, Japan, US; Countries included in asthma pooled OR: China, Thailand, Canada, Mexico, Madagascar, Spain, the Netherlands US; Countries included in chronic respiratory disease pooled OR: Hong Kong, China, Thailand, Canada, Mexico, Spain, the Netherlands, Germany, Japan, US; countries included in chronic cardiac disease pooled OR: Hong Kong, China, Thailand, Singapore, Canada, Mexico, Spain, the Netherlands, Germany, Japan, US; countries included in chronic liver disease pooled OR: Hong Kong, Thailand, Canada, Spain, the Netherlands, Japan; countries included in chronic neurologic disease pooled OR: China, Thailand, Singapore, Canada, Spain, the Netherlands, Japan, US; countries included in chronic renal disease pooled OR: Hong Kong, Thailand, Singapore, Canada, Mexico, Spain, Japan, US; countries included in diabetes pooled OR: Hong Kong, China, Thailand, Singapore, Canada, Mexico, Spain, Japan, US; countries included in immune compromised pooled OR: Hong Kong, China, Thailand, Singapore, Canada, Mexico, Spain, the Netherlands, Germany, Japan, US; countries included in obesity (≥30 BMI) pooled OR: China, Thailand, Spain, the Netherlands, US; countries included in pregnancy pooled OR: China, Thailand, Canada, Germany, US.

References

1. National Center for Immunization and Respiratory Diseases (2009) CDC. Use of Influenza A (H1N1) 2009 Monovalent Vaccine. Recommendations of the Advisory Committee on Immunization Practices (ACIP), 2009. MMWR 58: 1-8.
2. Fiore A, Shay D, Broder K, Uyeki T, Mootrey G, et al. (2009) Prevention and Control of Influenza, Recommendations of the Advisory Committee on Immunization Practices (ACIP), 2008. MMWR 57: 1-60.
3. Mereckiene J, Cotter S, Nicoll A, Levy-Bruhl D, Ferro A, et al. (2008) National seasonal influenza vaccination survey in Europe, 2008. Euro Surveill 13: pii: 19017.
4. Shimada T, Gu Y, Kamiya H, Komiya N, Odaira F, et al. (2010) Epidemiology of influenza A(H1N1)v virus infection in Japan, May - June 2009. Euro Surveill 14: pii=19244. Available online: http://www.eurosurveillance.org/ViewArticle.aspx?ArticleId=19244.
5. Poggensee G, Gilsdorf A, Buda S, Eckmanns T, Claus H, et al. (2010) The first wave of pandemic influenza (H1N1) 2009 in Germany: From initiation to acceleration. BMC Infectious Diseases 10: 155. doi:110.1186/1471-2334-1110-1155.
6. van 't Klooster T, Wielders C, Donker T, Isken L, Meijer A, et al. (2010) Surveillance of hospitalisations for 2009 pandemic influenza A(H1N1) in the Netherlands, 5 June - 31 December 2009. Euro Surveill 15: pii: 19461.
7. Hahné S, Donker T, Meijer A, Timen A, van Steenbergen J, et al. (2009) Epidemiology and control of influenza A(H1N1)v in the Netherlands: the first 115 cases. Euro Surveill 14: pii=19267. Available online: http://www.eurosurveillance.org/ViewArticle.aspx?ArticleId=19267.
8. Vriend HJ, Hahné SJM, Donker T, Meijer A, Timen A, et al. (2009) De nieuwe influenza A(H1N1)-epidemie in Nederland. Epidemiologische gegevens over de periode 30 april-14 augustus 2009. Ned Tijdschr Geneeskd 153: A969.
9. van der Sande MAB, van der Hoek W, Hooiveld M, Donker GA, van Steenbergen JE, et al. (2009) Bestrijding van de nieuwe influenza A (H1N1). II. Epidemiologie en niet-medicamenteuze maatregelen. Ned Tijdschr Geneeskd 153: A771.
10. Dijkstra F, van ’t Klooster TM, Brandsema P, van Gageldonk-Lafeber AB, Meijer A, et al. (2010) Jaarrapportage surveillance respiratoire infectieziekten 2009. Bilthoven: Rijksinstituut voor Volksgezondheid en Milieu, 2010. RIVM RIVM-briefrapportnummer: 210231006. Available from: <http://www.rivm.nl/bibliotheek/rapporten/210231006.pdf>.
11. INVS (2009) Surveillance de la grippe A (H1N1) 2009 en France : outils et méthodes. 16 décembre 2009. Available from: <http://www.invs.sante.fr/surveillance/grippe_dossier/docs_professionnels/methodo_surveillance_grippe_161209.pdf>.
12. Skarbinski J, Jain S, A B, Lee EJ, Huang J, et al. (*in press*) Hospitalized Patients with 2009 Pandemic Influenza A (H1N1) Virus Infection in the United States - September-October 2009.
13. Jain S, Kamimoto L, Bramley AM, Schmitz AM, Benoit SR, et al. (2009) Hospitalized Patients with 2009 H1N1 Influenza in the United States, April-June 2009. N Engl J Med 361: 1935-1944.

14. UK Health Protection Agency (2010) Sources of UK flu data Influenza Surveillance in the United Kingdom. Available at: http://www.hpa.org.uk/webw/HPAweb&HPAwebStandard/HPAweb_C/1195733821514?p=1191942171484.
15. Randrianasolo L, Raoelina Y, Ratsitorahina M, Ravolomanana L, Andriamandimby S, et al. (2010) Sentinel surveillance system for early outbreak detection in Madagascar. BMC Public Health 10.
16. Cutter J, Ang LW, Lai FY, Subramony H, Ma S, et al. (2010) Outbreak of pandemic influenza A (H1N1-2009) in Singapore, May to September 2009. Ann Acad Med Singapore 39: 273-282.
17. Sierra Moros MJ, Vázquez Torres M, Santa-Olalla Peralta P, Limia Sánchez A, Cortes García M, et al. (2010) Epidemiological surveillance activities during the 2009 influenza pandemic in Spain. Lessons learnt one year after. Rev Esp Salud Publica 84: 463-480.
18. Santa-Olalla Peralta P, Cortes-Garcia M, Vicente-Herrero M, Castrillo-Villamandos C, Arias-Bohigas P, et al. (2010) Risk factors for disease severity among hospitalised patients with 2009 pandemic influenza A (H1N1) in Spain, April - December 2009. Euro Surveill 15: pii=19667.
19. Santa-Olalla Peralta P, Cortes García M, Limia Sánchez A, Andrés Prado J, Pachón del Amo I, et al. (2010) Critically ill patients with 2009 pandemic influenza A (H1N1) infection in Spain: Factors associated with death, April 2009 - January 2010. Rev Esp Salud Publica 84: 547-568.
20. Baker MG, Wilson N, Huang QS, Paine S, Lopez L, et al. (2009) Pandemic influenza A(H1N1)v in New Zealand: the experience from April to August 2009. Euro Surveill 14.
21. Huang QS, Bandaranayake D, Lopez LD, Pirie R, Peacey M, et al. (2009) Surveillance for the 2009 pandemic influenza A (H1N1) virus and seasonal influenza viruses - New Zealand, 2009. MMWR Morb Mortal Wkly Rep 58: 918-921.
22. Yu H, Liao Q, Yuan Y, Zhou L, Xiang N, et al. (2010) Effectiveness of oseltamivir on disease progression and viral RNA shedding in patients with mild pandemic 2009 influenza A H1N1: opportunistic retrospective study of medical charts in China. BMJ 341.
23. Yu H (*in press*) Risk Factors for Severe Illness with 2009 Pandemic Influenza A(H1N1) Virus Infection in China. CID.
24. Campbell A, Rodin R, Kropp R, Mao Y, Hong Z, et al. (2010) Risk of severe outcomes among patients admitted to hospital with pandemic (H1N1) influenza. CMAJ 182: 349-355.
25. Helferty M, Vachon J, Tarasuk J, Rodin R, Spika J, et al. (*in press*) Canadian Pandemic H1N1 Cases: a Description of the Changing Epidemiology of Hospitalizations and Deaths in the First and Second Waves. CMAJ.
26. Kong WM (2000) A Review on the Hong Kong Influenza Surveillance System. Public Health & Epidemiology Bulletin 9.
27. Archer B, Cohen C, Naidoo D, Thomas J, Makunga C, et al. (2009) Interim report on pandemic H1N1 influenza virus infections in South Africa, April to October 2009: epidemiology and factors associated with fatal cases. Euro Surveill 14: pii: 19369.
28. Australian Government Department of Health and Aging (2010) Australian Influenza Surveillance Report No. 8, 2010. http://www.healthemergency.gov.au/internet/healthemergency/publishing.nsf/Content/EB136394E79CA5E2CA2576A50010783A/$File/ozflu-no8-2010.pdf.
29. Robert Koch Institute (2006) Data about health conditions in the general population/different age groups from a telephone survey (09/2003 - 03/2004) with 7341 participants. Available at:

http://www.rki.de/cln_171/nn_204490/DE/Content/GBE/Gesundheitsberichterstattung/GBEDownloadsB/gstel04,templateId=raw,property=publicationFile.pdf/gstel04.pdf.
30. Gommer AM, Poos MJJC, Hoeymans N (2001) Verloren levensjaren, ziekte en ziektelast voor 56 geselecteerde aandoeningen. In: Volksgezondheid Toekomst Verkenning, Nationaal Kompas Volksgezondheid. Bilthoven: RIVM, <<http://www.nationaalkompas.nl/>> Nationaal Kompas Volksgezondheid\Gezondheid en ziekte\Sterfte, levensverwachting en DALY's\Ziektelast in DALY's, 22 maart 2010.
31. Hoeymans N, Melse JM, Schoemaker CG (2010) Gezondheid en determinanten. Deelrapport van de VTV 2010 Van gezond naar beter. Bilthoven: Rijksinstituut voor Volksgezondheid en Milieu, 2010. RIVM report 270061006. Available from: <http://www.vtv2010.nl/object_binary/o9228_RIVM02-Gezondheid-en-determanten-VTV-2010.pdf>.
32. Instituto Nacional de Estadistica (2010) Population now-casts based on the 2001 census. Madrid: Instituto Nacional de Estadistica. Available from: <http://www.ine.es/jaxi/menu.do?type=pcaxis&path=%2Ft20%2Fp259&file=inebase&L>.
33. Instituto Nacional de Estadistica (2010) European Health Survey 2009. Results Preview. Madrid: Instituto Nacional de Estadistica. Available from: <http://www.ine.es/jaxi/tabla.do?path=/t15/p420/a2009/avance/l0/&file=01003.px&type=pcaxis&L=0>.
34. Aranceta J, Pérez Rodrigo C, Serra Majem L, Ribas Barba L, Quiles Izquierdo J, et al. (2003) Prevalence of obesity in Spain: results of the SEEDO 2000 study. Med Clin (Barc) 120: 608-612.
35. Hanslik T, Boelle P, Flahault A (2010) Preliminary estimation of risk factors for admission to intensive care units and for death in patients infected with A(H1N1)2009 influenza virus, France, 2009-2010. PLoS Curr Influenza 2010 Mar 9:RRN1150.
36. Japanese Ministry of Health Labor and Welfare (2005) Japan National Census 2005. Available at: <http://www.e-stat.go.jp/SG1/estat/GL08020101.do?_toGL08020101_&tstatCode=000001007251&requestSender=search> and http://www.stat.go.jp/english/data/kokusei/index.htm.
37. Japanese Ministry of Health Labor and Welfare (2005) Patients Survey in 2005. Available at: http://www.mhlw.go.jp/english/database/db-hss/ps.html.
38. Statistics Canada (2009) Canadian Community Health Survey. The Daily: Thursday June 25, 2009. Available at: <http://www.statcan.gc.ca/daily-quotidien/090625/dq090625b-eng.htm>.
39. Census and Statistics Department Hong Kong Special Administrative Region (2006) Statistical Tables of Population by Age Group and Sex. Available from: <http://www.censtatd.gov.hk/hong_kong_statistics/statistical_tables/index.jsp?charsetID=1&tableID=002>
40. Surveillance and Epidemiology Branch Centre for Health Protection Department of Health Hong Kong SAR (2009) Behavioural Risk Factor Survey (April 2009), Section 3.2:Weight status and control, page 21. Available from: <http://www.chp.gov.hk/files/pdf/brfs_april_2009_eng.pdf>
41. Census and Statistics Department Hong Kong Special Administrative Region (2008) Special Topics Report No.48: Persons with disabilities and chronic diseases. Persons with chronic diseases by selected type of chronic diseases, page 90. Available from: <http://www.censtatd.gov.hk/products_and_services/products/publications/statistical_report/social_data/index_cd_B1130148_dt_latest.jsp>

42. Department of Health of Hong Kong Special Administrative Region and Department of Community Medicine of the University of Hong Kong (2004) Population Health Survey 2003/2004, Chatper 4: Physical Health, page29. Available from: <http://www.chp.gov.hk/files/pdf/full_report_on_population_health_survey_2003_2004_en_20051024.pdf>
43. National Bureau of Statistics of China (2009) China Statistical Yearbook 2009. Beijing: China Statistics Press.
44. Zhong NS, Wang C, Yao WZ, Chen P, Kang J, et al. (2007) Prevalence of Chronic Obstructive Pulmonary Disease in China- A Large, Population-based Survey. Am J Respir Crit Care Med 176: 753-760.
45. National cooperation group on childhood asthma (2003) A nationwide survey in China on prevalence of asthma in urban children. Chin J Pediatr 41: 123-127.
46. Wang GB, Pen YL, Du CM, Tang BX, Liu JG, et al. (2002) Epidemiological survey on bronchial asthma in Henan province Chin J Tuberc Respir Dis 25: 25-28
47. Tang TQ, Ding Y, Zheng JP, Wang XP, Ma QF, et al. (2000) Epidemiological survey and analysis on bronchial Asthma in Guangdong province. Chin J Tuberc Respir Dis 23: 730-733.
48. Chen P, Yu RH, Hou XM, Tan PQ, Xie H, et al. (2000) Epidemiological survey on bronchial asthma in Liaoning province. Chin J Tuberc Respir Dis 25: 730-733.
49. Cao BY, Mi J, Gong CX, Cheng H, Yan C, et al. (2007) The prevalence of diabetes in Children and adolescents of Beijing Chin J Epidemiol 2007 28: 631-634.
50. Chinese Diabetes Society (2008) The prevalence of diabetes and metabolic syndrome of 14 provinces in China, 2008. Available from: <http://www.diab.net.cn/page.jsp?id=15>
51. Jiang LH, Duan CQ, Ma ZQ (2004) Study progress on the epidemiological investigation on the congenital heart disease West China Med J 19: 510-511.
52. Ministry of Health of People's Republic of China, editor (2009) 2009 year book of health in the People's Republic of China: People's Medical Publishing House.
53. Liu B, Gao ES (2002) Risk Factors for Spontaneous Abortion of Chinese Married Women at Reproductive Age. Chin J Public Health 18: 890-892.
54. Jamieson DJ, Honein MA, Rasmussen SA, Williams JL, Swerdlow DL, et al. (2009) H1N1 2009 influenza virus infection during pregnancy in the USA. The Lancet 374: 451-458.
55. Chen CM (2008) Overview of obesity in Mainland China. Obes Rev 9 Suppl 1: 14-21.
56. Wang LD, editor (2005) Comprehensive Report, Chinese nutrition and health survey in 2002: People's Medical Publishing House. 50-52 p.
57. World Health Organization (2009) Global tuberculosis control: a short update to the 2009 report. Available from: <http://www.who.int/tb/publications/global_report/2009/update/en/>
58. United Nations (2009) United Nations Population Division. World Population Prospects: The 2008 Revision Population Database. http://esa.un.org/unpp/.
59. Higgins J, SG T (2002) Quantifying heterogeneity in a metaanalysis. Stat Med 21: 1539-1558.
